# The genetically encoded biosensor FEOX is a molecular gauge for cellular iron environment dynamics at single cell resolution

**DOI:** 10.1038/s41598-025-20428-5

**Published:** 2025-10-21

**Authors:** Carolyn Sangokoya

**Affiliations:** 1 Department of Pathology, University of California San Francisco, San Francisco, CA USA; 2 The Eli and Edythe Broad Center of Regeneration Medicine and Stem Cell Research, University of California San Francisco, San Francisco, CA USA

**Keywords:** Embryonic stem cells, Sensors and probes, Genetic engineering

## Abstract

Iron-sensing is evolutionarily conserved among life on Earth, and mammalian cells are known to sense cellular iron through mechanisms similar to bacteria and marine invertebrates. While iron regulatory proteins (IRPs) function as RNA-binding proteins during iron-limiting conditions and reflect cellular iron status, we lack genetically-encodeable tools to quantify bioavailable intracellular iron status over developmental time and at single-cell resolution. In order to better understand the cellular environment that supports or restricts IRP-active conditions, particularly during complex and dynamic changes leading to differentiated states, we present a ratiometric genetically-encoded biosensor called FEOX. FEOX is a ratiometric fluorescent biosensor of the cellular iron environment based on a mammalian hemerythrin-like domain, acting as an iron-dependent ligand-based regulatory switch. Compared to increased IRP activity in response to iron-limitation, FEOX dynamics demonstrate decreased ratiometric fluorescence representing decreased cellular iron during iron-limitation. Using FEOX we are able to quantify the dynamics of the bioavailable cellular iron environment during early stem cell differentiation by ratiometric measurements at single-cell resolution. Results from FEOX and from an IRP activity sensor during stem cell pluripotency transition and early differentiation provide orthogonal support for an increased iron demand state. Using these genetically-encodeable tools will allow greater insight into cellular iron homeostasis within mammalian systems. IRPs function as RNA-binding proteins during iron-limiting conditions, regulating the stability and translation of messenger RNAs that are essential for cellular iron homeostasis. However, to better understand and study the cell and molecular regulators of the iron-limiting conditions required for IRP RNA-binding activity, new tools are needed to monitor iron-responsive alterations in the intracellular environment at single-cell resolution and over developmental time. We devised a genetically-encoded biosensor called FEOX that depends on molecular iron interactions independent of IRP function and directly gauges bioavailable cellular iron.

**Supplementary Information:**

The online version contains supplementary material available at 10.1038/s41598-025-20428-5.

## Introduction

In mammalian cells, activity of the iron regulatory proteins (IRPs) reflects cellular iron status^1^. IRPs function as RNA-binding proteins during iron-limiting conditions, regulating the stability and translation of messenger RNAs encoding proteins essential for cellular iron homeostasis. However, the system by which cells sense and gauge bioavailable cellular iron depends on iron binding at the evolutionarily conserved iron-binding hemerythrin-like (Hr) domain of the F-box/LRR-repeat protein 5 (FBXL5)^2,3^. FBXL5 senses intracellular iron levels via iron binding to its N-terminal hemerythrin-like domain^2,3^. In iron replete conditions, iron binding stabilizes FBXL5 and enables its assembly into the multi-subunit E3 ubiquitin ligase complex with SKP1, RBX1, and CUL1 that catalyzes the ubiquitin-dependent degradation of IRP2. In iron deplete conditions, FBXL5 is itself susceptible to degradation via the ubiquitin-proteasome pathway, resulting in stabilization of IRP2^2,3^. and its subsequent ability to function as an RNA-binding protein and regulator of iron homeostasis To better understand and study functional molecular regulators of the iron-limiting conditions required for IRP RNA-binding activity, new tools are needed to monitor iron-responsive alterations in the intracellular environment at single-cell resolution, and to follow these responses over developmental time. To accomplish this, we have devised a genetically-encoded iron-sensing sensor called FEOX that is based on the FBXL5 hemerythrin-like domain, as that domain acts as a ligand-dependent regulatory switch mediating FBXL5 stability.

**Figure 1:**
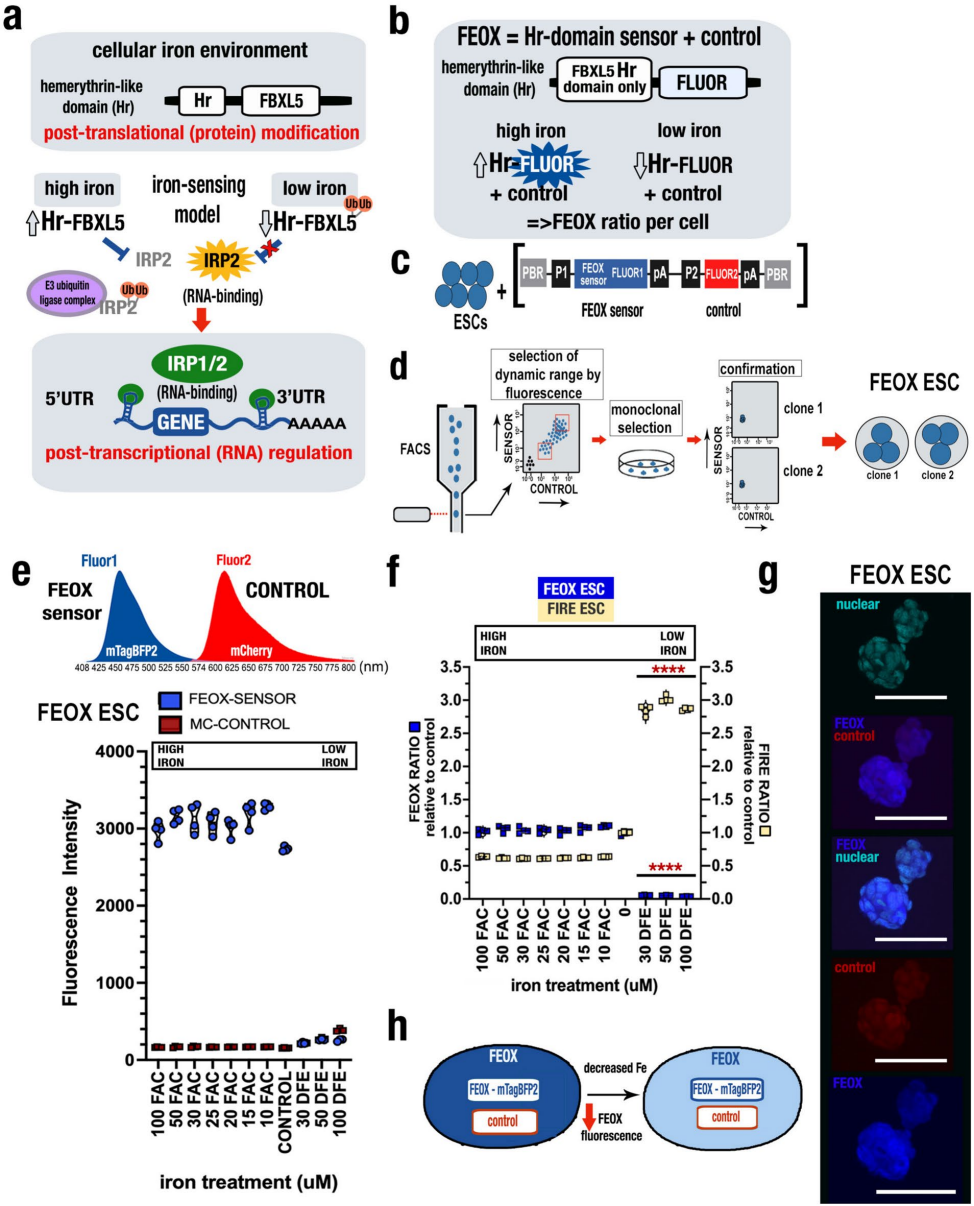
FEOX is a ratiometric biosensor of the cellular iron environment (a) Schematic describing regulatory phases of cellular iron homeostasis: iron-sensing in the cellular iron environment is driven by the hemerythrin-like domain of FBXL5 (top); In differential iron states the response at this domain leads to post-translational protein modification (ubiquitination) that shape iron responses by controlling IRP2 accessibility for RNA-binding (middle) and further post-transcriptional RNA regulation of IRP target transcripts (bottom). **(b)** Schematic depicting the encoded synthetic gene FEOX formed by the Hr domain of FBXL5 connected to a gene encoding a fluorescent protein **(c)** Schematic map organization of FEOX biosensor showing the FEOX sensor and control cassettes. PBR=*piggyBac* transposon repeat sequence; P1/P2= promoter; FLUOR= fluorescent protein open reading frame; pA=poly-adenylation sequence. **(d)** Diagram of workflow from transfected embryonic stem cells (ESC) to selection by fluorescence-activated cell sorting (FACS), monoclonal selection and confirmation of clonality by flow cytometry. **(e)** Schematic depicting emission spectra for Fluor1 (mTagBFP2) and Fluor2 (mCherry) (top). Iron gradient assay for high iron treatment by ferric ammonium citrate (FAC), and low iron treatment by iron chelator deferoxamine (DFE), compared to untreated condition. **(f)** Graph showing overlay of iron gradient experiments with FEOX ESC (blue) and with FIRE ESCs (yellow), respectively. FEOX ratio (ratiometric fluorescence intensity Fluor1 to Fluor2) relative to untreated condition on left y-axis. FIRE ratio (ratiometric fluorescence intensity Fluor1 to Fluor2) relative to untreated condition on right y-axis. FEOX ratio data is normalized data from data shown in (e). Mean measurements per experiment for each gradient condition (n=4) are shown. Biological replicates are shown. Error bars represent SEM. ****P<0.0001, unpaired two-tailed t test. **(g)** Microscopic images of FEOX ESCs growing in colonies, demonstrating (top to bottom) nuclear stain (cyan) alone; merged endogenous TagBFP2 (FEOX sensor, blue) and mCherry (control, red) fluorescence; merged endogenous TagBFP2 (FEOX sensor, blue) and nuclear stain (cyan); mCherry (control, red) alone; and TagBFP2 (FEOX sensor, blue) alone. Scale bar = 90 μm.** (h)** Schematic of cell with FEOX ratiometric sensor both with (left) and without (right) stabilization by high iron concentration, where decreased iron alters stability and illumination of the FEOX sensor fluorescent protein secondary to the cellular iron environment.

The FBLX5 Hr domain is related to hemerythrin domains found in bacteria and marine invertebrates containing di-iron centers that reversibly bind iron and oxygen^4–6^. However while the majority of described hemerythrins reversibly bind oxygen at the di-iron center^4–7^, the FBXL5 Hr domain does not bind oxygen either in isolation or in the context of the full FBXL5 protein^8,9^. Structural studies of this domain reveal its di-iron center as the basis for iron sensing, a feature distinctly present on FBXL5, even when compared to other F-box proteins^3,7–9^. Previous studies of this domain have detailed a selectivity for responsiveness to iron in comparison with other metals, including cobalt, zinc, manganese, nickel, copper, and magnesium^8^. These features make the isolated FBXL5 Hr an ideal substrate for cellular iron-sensing studies. FEOX is designed as a heterologous synthetic protein using the human FBXL5 Hr domain (with the features as described above) fused to a fluorescent protein. As with FBXL5, the FEOX hemerythrin-like domain confers iron-dependent regulation of stability. The premise of FEOX is that the loss of iron needed by the hemerythrin-like FBXL5 domain renders this engineered synthetic protein susceptible to proteasomal degradation^2^, with functionality by fluorescence. Together with the recent FIRE biosensor that is sensitive to IRP-binding activity^10^, with FEOX we are now able to provide complementary IRP-independent orthogonal data about the cellular iron environment supporting the IRP activity and cellular iron homeostasis at single-cell resolution. In the cellular environment of mammalian cells, iron-sensing occurs through the di-iron center of the hemerythrin-like domain of the FBXL5 protein^2,8,9,11^. The high/replete-iron condition results in stabilized FBXL5 protein that subsequently promotes IRP2 polyubiquitination and degradation, while the low iron condition results in polyubiquitination and degradation of FBXL5 itself, leading to subsequent stabilization of IRP2 (Fig. 1a). IRP RNA-binding activity by both IRP1 and IRP2 result in binding to untranslated regions (UTR) of targeted transcripts and the translational repression (5’UTR) or stabilization (3’UTR) of the transcript via post-transcriptional RNA regulation (Fig. 1a). The IRP activity sensor FIRE takes advantage of this setup by using IRP binding sites in the 3’UTR of a synthetic gene encoding a fluorescent protein, such that IRP1/2 can bind to, stabilize the transcript, and fluoresce with IRP activity^10^.

## Results

### FEOX is a ratiometric biosensor of the cellular iron environment based on a mammalian hemerythrin-like domain

To engineer the FEOX sensor, two cassettes (sensor and control) were designed, each encoding a synthetic gene composed of a fluorescent protein driven by a ubiquitous mammalian promoter and punctuated by a poly-adenylation region. The FEOX sensor cassette specifically encodes a synthetic hemerythrin-like domain in frame with a fluorescent protein (Fig. 1b, Fig. S1). The sensor and control cassettes are flanked by *piggyBac* transposon regions such that the cassettes are incorporated together into the genome (Fig. 1cf). Genomic integration by transfection into mouse embryonic stem cells (ESCs) and validation by flow cytometry allows for sensitive selection of the desired dynamic range of ratiometric expression of fluorescence in transfected cells. Flow sorting and monoclonal selection followed by additional cytometric confirmation leads to the generation of FEOX ESC clones that express the FEOX (mTagBFP2-sensor, mCherry-control) fluorescent proteins (Fig. 1d-e).

To test the response of these FEOX-encoded cells in iron-deplete and iron-replete conditions, the cells were subject to iron gradients by iron chelation (low iron) or addition (high iron), then assayed for fluorescence by flow cytometry. The FEOX sensor demonstrated dramatically decreased fluorescence in response to iron chelation and modestly increased fluorescence in response to iron addition (Fig. 1e-f). These findings are also shown by FEOX Ratios -- ratiometric quantitation based on the ratio of sensor to control fluorescence intensities per cell (Fig. 1f). Comparatively, when FIRE (IRP activity sensor)-encoded ESCs^10^ are subjected to iron chelation, the FIRE sensor demonstrated dramatically increased fluorescence in response to iron chelation and decreased fluorescence in response to iron addition, with FIRE Ratios significantly higher in low iron conditions (Fig. 1f). Imaging of FEOX ESCs demonstrates predominantly cytoplasmic intracellular fluorescence of the FEOX sensor and control, with additional control fluorescence seen in the nucleus (Fig. 1g, Fig. S1). In summary, the FEOX sensor fluorescence (and subsequently FEOX Ratio) is decreased in low iron conditions, a readout of decreased FEOX fluorescent protein stability (Fig. 1h).

Our iron gradient studies in undifferentiated ESCs showed a modest (up to 11%) rise in the FEOX ratio when tested with exogenous iron. This decreased sensitivity of FEOX sensor mirrors that of previous studies of a restricted ability of the isolated FBXL5-Hr domain to respond to exogenous elevation in bioavailable iron when tested in the human HEK293 cell line^8^. In the context of assaying cell response to supraphysiologic iron levels we note that in its current form FEOX is most appropriate for use under physiologic levels of iron, with a much greater capacity to identify sensitivity and dynamics of iron deficiency, and that the dynamic range of FEOX ratios in the studied cell type/system must be determined (as in Fig. 1e-f).

### Ratiometric quantitation by FEOX illuminates the dynamics of the cellular iron environment during early ESC differentiation at single-cell resolution

**Figure 2:**
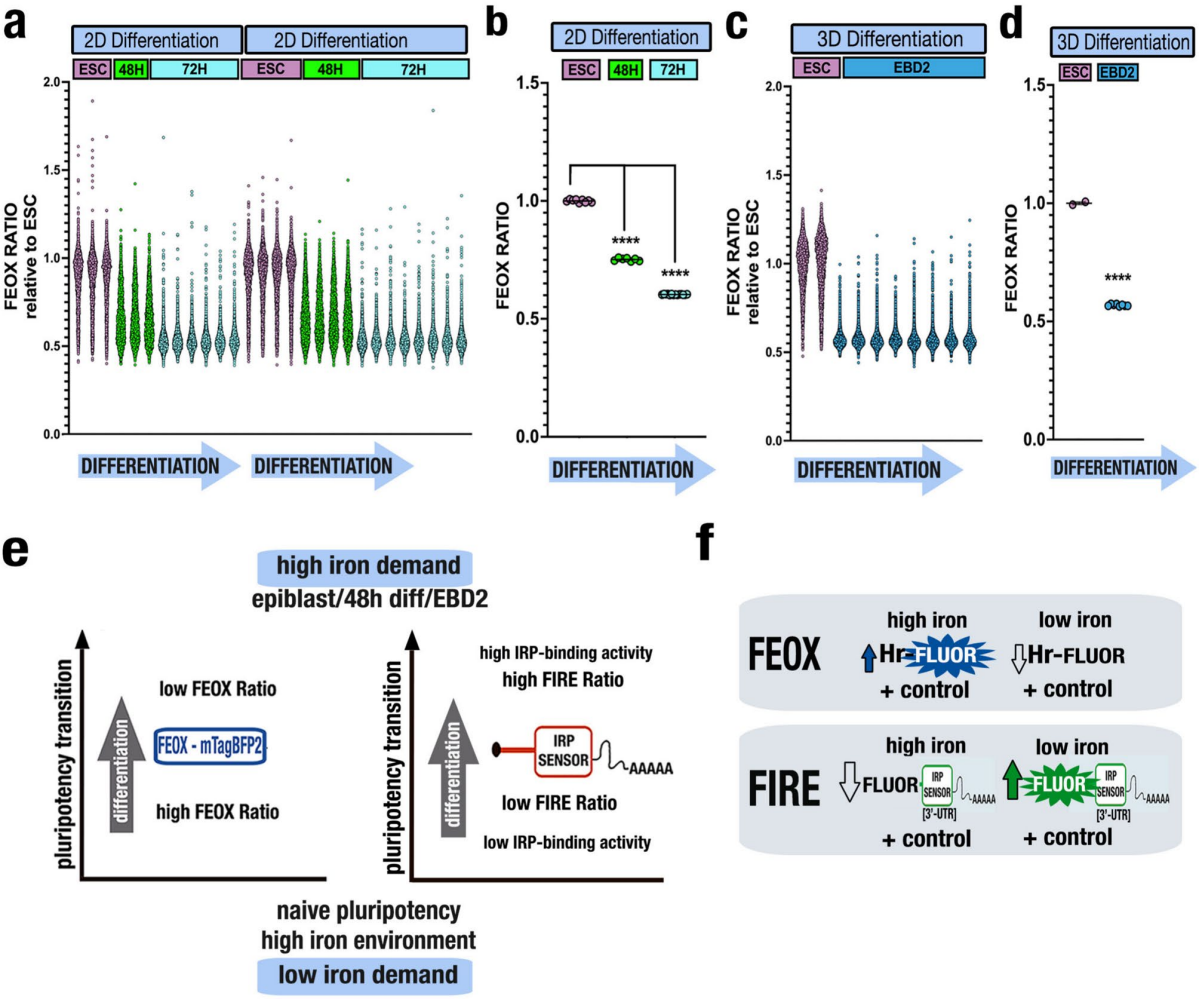
FEOX quantifies dynamics of the cellular iron environment during early ESC differentiation at single-cell resolution (**a**) Flow cytometry analysis of FEOX ESCs in naïve state and after 48 and 72 hours 2D differentiation. FEOX Ratios (sensor to control intensity values per cell) relative to ESC median (ESC n= 20616 cells, 48H=20356 cells, 72H n=36062 cells). Biological replicates are shown for n=21 differentiation experiments. 2D = two dimension **(b)** Mean FEOX Ratios per experiment in **(a).** Error bars represent SEM. ****P<0.0001, unpaired two-tailed t test. **(c)** Flow cytometry analysis of FEOX ESCs and embryoid bodies at EBD2. FEOX Ratios (sensor to control intensity values per cell) relative to ESC median (ESC n= 9979 cells, EBD2 n= 134162 cells). Biological replicates are shown for n=8 differentiation experiments. 3D = three dimension. **(d)** Mean FEOX Ratios per experiment in **(c).** Error bars represent SEM. ****P<0.0001, unpaired two-tailed t test. **(e)** Schematic summary of experimental results of FEOX Ratios and FIRE Ratios taken along the pluripotency transition from naïve (ESC) to formative state (epiblast/EBD2/48h) differentiation, in context of parallel relative iron demand and iron environment.** (f)** Schematic of parallel features of FEOX and FIRE sensors for the study of intracellular iron states.

Cellular iron regulation is essential for embryonic development^1,12–14^, yet we lack tools to interrogate the dynamics of the cellular iron environment over developmental time, particularly during the earliest stages of cell fate differentiation. To characterize the cellular iron environment using FEOX during ESC differentiation, we performed stem cell differentiation in FEOX-encoded ESCs by LIF/2i removal from ESC culture for 48 hours (Epi-like cell transition) and 72 hours (early stage differentiation). As many labs in this field have both two-dimensional (2D) and three-dimensional (3D) modes of ESC culture, we explored the dynamics of FEOX in both 2D ESCs and 3D stem cell-based embryoid bodies. In 2D ESC culture, FEOX Ratios decreased progressively and significantly between ESCs at both the 48 hour and 72 hour differentiation timepoints (Fig. 2a-b, Fig. S2). FEOX Ratios, shown per cell relative to ESC, demonstrated distinct downward shifts during pluripotency transition and early differentiation, indicating decreased intracellular iron levels (Fig. 2a-b, Fig. S2). Similar results were seen in ESC culture to 3D embryoid body differentiation for 48 hours (EBD2).

The FEOX sensor reveals dynamics of the cellular iron environment during stem cell transition from naïve pluripotency to the epiblast-like state and early differentiation. The relatively high FEOX Ratio in naïve pluripotency corresponds with high iron and low IRP-binding activity in FIRE-encoded cells^10^ at the same stage. Upon pluripotency transition and differentiation, the decreased FEOX Ratios reveal decreased cellular iron and confirm the iron-limiting condition necessary for the documented increased IRP-binding activity seen at the same stages in FIRE-encoded cells^10^ (Fig. 2e). If used in parallel, both FEOX and FIRE are complementary tools with potential to provide valuable information about the intracellular iron state over developmental time (Fig. 2f).

## Discussion

To date, the development of fluorescent iron probes has increased our ability to study cellular iron status^15–19^, however the design of this FEOX sensor utilizes both the isolated FBXL5 hemerythrin domain’s specific iron-sensing capability and its iron-dependent stabilization and degradation dynamics while enabling incorporation into mammalian cells as a ratiometric genetically-encoded cassette reporter system. The approach presented here using *piggyBac* transposon-based integration of FEOX and flow cytometry for user-controlled selectivity preferentially takes advantage of a non-viral method of robust integration with single-cell capacity for controlling the preferred level of integration and for testing the functional dynamic range of the sensor. Here for the first time, we illustrate this system in pluripotent cells (mouse embryonic stem cells) that are capable of differentiation into cells from all three germ layers. Using this sensor, we demonstrate the ability to test the physiologic capacity of the cellular iron environment through gradients as well as to follow endogenous physiologic and developmental changes of the cell through the lens of the cellular iron environment.

FEOX highlights the dynamics of the cellular iron environment during early stem cell differentiation. Evaluation of FIRE^9^ and FEOX sensors in both 2D and 3D stem cells culture environments and in mammalian cells can yield orthogonal data to quantify, identify, and illuminate core aspects of cellular iron homeostasis as IRP-dependent and IRP-independent (respectively) mechanisms during complex processes and over developmental time. The summarizing data for both sensors in this developmental window supports a cell state of transitioning low cellular iron with increasing high IRP activity, high iron demand during early differentiation. It is intriguing that the as-yet-unknown targets of high IRP activity at this stage could play roles in metabolic and lineage priming towards cell states and fates.

The core aspects of cellular iron homeostasis: iron import, export, storage, and utilization, as well as the roles for iron as core elements within proteins, in complex within heme, and as iron-sulfur clusters, are essential for most cellular functions including ATP generation, DNA metabolism, mitochondrial function, and oxygen transport^1,20^. While IRP-dependent mechanisms are critical, quantifying the cellular iron environment with rigorous ratiometric measurement by FEOX allows the capability for a broader, complementary readout of iron-responsive effects in a wide range of cellular processes to better understand the cellular environment that supports or restricts IRP-active binding conditions. Additionally, FEOX contributes to building platforms to dissect the additional and synergistic contributions of oxygen, pH levels, and other metals that work with iron in different ways in different cell types. As metabolic assessments become integral for the parallel and subsequent epigenetic and epi-transcriptomic assessments of cell states, the ability to follow intracellular iron status over time with FEOX will add core insights to basic and complex functions of diverse cell types.

FEOX provides a readout at the single-cell-level, a limitation as compared to more selective probes that can localize to organelles or report on ferric or ferrous iron states^21,22^. FEOX also is restricted in its response to exogenous or supraphysiologic iron, and requires a functional mammalian ubiquitin-proteasome system. Despite these limitations, the strengths of the genetically-encoded cell level readout of the intracellular iron environment will be best utilized in metabolically or developmentally active environments where cell fates and states are dynamic. In the presence of IRP activity information, FEOX can add environmental context, and in the absence of IRP activity information, the FEOX status can offer a ratiometric (relative) readout between cell states. We expect that FEOX offers opportunities to multiplex with other specific sensors including FIRE to further discover and dissect molecular-metabolic networks at single-cell resolution over time.

## Methods

### Experimental model and study participant details

#### ESC cell culture

V6.5 mouse ESCs (RRID:CVCL_C865) derived from the F1 hybrid of 129SvJae/C57BL/6 and FIRE1CS-V6.5 ESCs (RRID:CVCL_E6HQ) were grown and maintained in the Sangokoya Laboratory at 37C and 5%CO2 on 0.2% gelatin-coated plates, cultured in ESC medium (custom DMEM) supplemented with 15% FBS (Corning Mediatech), LIF (1000U/mL, custom) and 2i (1uM PD0325901 (Axon MedChem) and 3uM CHIR99021 (Axon MedChem).

### Method details

#### Construction and cloning of FEOX cassettes

DNA oligonucleotides and synthetic gene fragments were obtained from Integrated DNA Technologies (IDT). All constructed plasmids are available on Addgene. All vectors for mammalian cell experiments were purified using ZymoPURE II Plasmid Midiprep kits (Zymo Research). For FEOX, **FEOX-PBCS-TBF2-MC2** containing the sensor and control cassettes was constructed, synthesized, and cloned as in the Fig 1 schematic. The FEOX sensor cassette, under control of the EF-1α -alpha promoter, encodes the fluorescent protein mTagBFP2^23^ and sensor composed of a 161 amino acid sequence (Fig. S1) synthesized based on known mammalian FBXL5 hemerythrin-like site (Fig. S1). The control cassette, under control of the human phosphoglycerate kinase promoter, was constructed, synthesized, and cloned as in Fig 1 schematic and encodes the fluorescent protein mCherry^24^.

#### Generation of FEOX ESCs

Transfection of mouse ESCs with the plasmids containing the FEOX sensor and control cassettes and *piggyBac* transposase was done using Fugene6 (Promega) 16 hours prior to fluorescence-activated cell sorting (FACS) on a FACSAria II SORP cytometer (Becton Dickinson) to assay transfected cells by fluorescence and collect preferred clones. Re-analysis and re-sorting of clones was performed within two passages to confirm stable transfection. After plating and selection of single clones and monoclonal culture, final assessment was performed by flow cytometry to confirm FEOX expression in single clones.

#### Cell culture and embryoid body generation and differentiation

Differential states of mouse ESC pluripotency from naïve to Epi-like cell transition were generated by removal of LIF and 2i. With 2D differentiation, this removal lasted for 48 hour or 72 hours. For 3D differentiation and generation of embryoid bodies, first ESCs were plated in ESC medium (as described above). To initiate differentiation, LIF and 2i were removed. Cells were collected at indicated time points following LIF/2i removal. For embryoid body (EB) generation, ESCs were cultured in rotary (100 rpm) suspension culture on a low attachment plate allowing for self-aggregation and regular reproducible spontaneous differentiation over an indicated course of days.

#### Flow cytometry

After dissociation into single-cell suspension using Trypsin or TrypLE (ThermoFisher Scientific), cells were resuspended in HBSS/1% BSA and filtered for size by cell strainer (35 micron mesh). Cells were processed by flow cytometry on an LSR II flow cytometer (Becton Dickinson), with events collected by FACS Diva software (BD Biosciences) using a hierarchical gating strategy selecting for a homogenous cell population (forward-scatter area vs. side-scatter area) of single cells (forward-scatter width vs. forward-scatter area). Dot plots and mean fluorescence intensities for gated events were generated using FlowJo analysis software (v10.9.0, www.flowjo.com) and GraphPad Prism (v10, www.graphpad.com).

#### Imaging

FEOX ESCs were grown on 0.2% gelatin-coated glass chamber slides, stained briefly with NucRed Live 647 ReadyProbes Reagent (Invitrogen), then fixed with 4% paraformaldehyde and imaged using the ECHO Revolve microscope.

### Quantification and statistical analysis

No data were excluded from the analyses. Statistical tests were performed in GraphPad Prism (v10). Data collection and analysis were not performed blind to the conditions of the experiments, but data analyses have been performed with identical parameters and software. The number of biological replicates and independent experiments, both equal to or greater than 3, is indicated in figure legends. The statistical tests used are indicated in the figure legends

## Supplementary Information

Supplementary Information.

## Data Availability

Source data supporting this study has been deposited at Zenodo at 10.5281/zenodo.15860525 and is publicly available as of the date of publication. - Plasmids generated in this study have been deposited at Addgene and are publicly available as of the date of publication. - This paper does not report original code. - Any additional information required to reanalyze the data reported in this paper is available upon reasonable request to the corresponding author.
