# Supplementary material for: The genetically encoded biosensor FEOX is a molecular gauge for cellular iron environment dynamics at single cell resolution
Source: Sci Rep. 2025 Oct 21;15:36596. doi: 10.1038/s41598-025-20428-5 (PMC12541023; doi:10.1038/s41598-025-20428-5)
Supplement: Supplementary file 1 — Supplementary Information. [file 41598_2025_20428_MOESM1_ESM.docx]

**Supplemental information**

Document S1. Figures S1 (related to Figure 1).

A.) FEOX sensor sequence illustrated within schematic of sensor cassette B.) gating strategy for flow cytometry after capture cells by side-scatter SSC-A vs forward-scatter FSC-A, use FSC-W (width) vs FSC-A to gate/subset single cells, then use negative control no-color cells to draw negative color gates, finally (in blue), sample FEOX cells depict positive color in the FEOX channels that read emissions for mTAGBFP2 (Violet F) and mCherry (YG-C), identifying dual-color cells. Two different differentiation stages (ESC on left, 72h differentiation on right) depicted as example data. C.) Images of undifferentiated FEOX ESCs growing as colonies at 20x (scale bar 90 microns) with fluorescence from FEOX sensor (blue-TAGBFP2), control fluor (red-MCherry), and cell-permeant nuclear stain (teal-647nm NucLiveReadyProbe).

Document S2. Figures S2 (related to Figure 2).

1. Median fluorescence intensity for FEOX channels mTAGBFP2 (blue) and mCherry (red), corresponding to

data in Figure 2B. B.) Median fluorescence intensity for FEOX channels mTAGBFP2 (blue) and mCherry (red), corresponding to data in Figure 2D.
